# Supplementary material for: Objective monitoring of loneliness levels using smart devices: A multi-device approach for mental health applications
Source: PLoS One. 2024 Jun 20;19(6):e0298949. doi: 10.1371/journal.pone.0298949 (PMC11189241; doi:10.1371/journal.pone.0298949)
Supplement: S1 Appendix — (PDF) [file pone.0298949.s001.pdf]

1 S Appendix

Table S 1. List of features extracted from smart ring

Category	Feature
Activity	Average activity level during the whole day.
	Number of kilocalories one spent in a day based on the amount of physical activity
	The total number of kilocalories one consumed in a day
	Every 5 minutes a character is shown to indicate the level of intensity of an activity.
	How many minutes of high intensity activity was performed in a day.
	Number of minutes where one is not doing anything in a day.
	Number of minutes where one is doing low intensity activity in a day.
	Number of minutes where one is doing medium intensity activity in a day.
	The average amount of energy it costs to complete a task for each minute of the span of one activity period.
	Total number of minutes of when the user was not wearing the ring during the day
	Total number of minutes spent sleeping during a day.
	Number of steps taken during the day
Sleep Features	the amount of time spent awake during your sleep period
	average beats per minute
	percentage of sleep period spent asleep
	heart rate at 5 minutes each sleep period
	average heart rate during the sleep period
	lowest heart rate during sleep period (resting heart rate)
	The time it takes a person to fall asleep after turning the lights out
	Percentage of sleep time when the user was moving
	Measure of the beat-to-beat differences in heart rate
	Measures the beat-to-beat differences in heart rate in the first five minutes of the sleep period
	The measure of the change in your body temperature while you sleep
	The difference between your body's core temperature and the temperature of your sleeping environment
	Represents the amount of time a person spends asleep during a given period of time

Table S 2. List of features extracted from smart watch

ID	Feature
HRV_MeanNN	The mean of the RR intervals.
HRV_SDNN	The standard deviation of the RR intervals.
HRV_SDANN1	The standard deviation of average RR intervals extracted from 1-minute segments of time series data
HRV_SDNNI1	The mean of the standard deviations of RR intervals extracted from 1-minute segments of time series data
HRV_RMSSD	The square root of the mean of the squared successive differences between adjacent RR intervals.
HRV_SDSD	The standard deviation of the successive differences between RR intervals.
HRV_CVNN	The standard deviation of the RR intervals (SDNN) divided by the mean of the RR intervals (MeanNN).
HRV_CVSD	The root mean square of successive differences (RMSSD) divided by the mean of the RR intervals (MeanNN).
HRV_MedianNN	The median of the RR intervals.
HRV_MadNN	The median absolute deviation of the RR intervals.
HRV_MCVNN	The median absolute deviation of the RR intervals (MadNN) divided by the median of the RR intervals (MedianNN).
HRV_IQRNN	The interquartile range (IQR) of the RR intervals.
HRV_PNN50	The proportion of RR intervals greater than 50ms, out of the total number of RR intervals.
HRV_PNN20	The proportion of RR intervals greater than 20ms, out of the total number of RR intervals.
HRV_HTI	The HRV triangular index, measuring the total number of RR intervals divided by the height of the RR intervals histogram.
HRV_TINN	The baseline width of the RR intervals distribution obtained by triangular interpolation, where the error of least squares determines the triangle. It is an approximation of the RR interval distribution.
HRV_LF	The spectral power of low frequencies (0.04 to 0.15 Hz).
HRV_HF	The spectral power of high frequencies (0.15 to 0.4 Hz).
HRV_VHF	The spectral power of very high frequencies (0.4 to .5 Hz).
HRV_LFHF	The ratio obtained by dividing the low frequency power by the high frequency power.
HRV_LFn	The normalized low frequency, obtained by dividing the low frequency power by the total power.
HRV_HFn	The normalized high frequency, obtained by dividing the low frequency power by the total power.
HRV_LnHF	The log transformed HF.
HRV_SD1	Standard deviation perpendicular to the line of identity. It is an index of short-term RR interval fluctuation
HRV_SD2	Standard deviation along the identity line. Index of long-term HRV changes.
HRV_SD1SD2	Ratio of SD1 to SD2. Describes the ratio of short term to long term variations in HRV.
HRV_S	Area of ellipse described by SD1 and SD2 (pi * SD1 * SD2)
HR	Heart rate

Table S 3. List of features extracted from smartphone

Category	Feature
Call	Outgoing call duration
	Income call counts
	Outgoing call counts
	Missedcall counts
	Voicemail counts
Notification	Number of notifications from applications type: Productivity
	Number of notifications from applications type: Photography
	Number of notifications from applications type: Communication
	Number of notifications from applications type: Lifestyle
	Number of notifications from applications type: Social
	Number of notifications from applications type: Shopping
	Number of notifications from applications type: Health & Fitness
	Number of notifications from applications type: Entertainment
	Number of notifications from applications type: Music & Audio
Messages	Number of received messages
	Number of sent messagess
Screen	Number of screen off
	Number of screen on
	Number of screen locks
	Number of screen unlocks
Location (GPS)	Variance of latitude
	Variance of speed
	Mean of speed
	Number of places
	Home duration
	Outdoor duration
	Mean of outdoor duration
	Standard deviation of outdoor duration
	Type of the place with longest duration other than home
	Total travel distance
Battery	Number of battery charger plugins
