# Supplementary material for: Objective monitoring of loneliness levels using smart devices: A multi-device approach for mental health applications
Source: PLoS One. 2024 Jun 20;19(6):e0298949. doi: 10.1371/journal.pone.0298949 (PMC11189241; doi:10.1371/journal.pone.0298949)
Supplement: Attachment — Submitted filename: PLOS Human Participants Research Checklist.pdf [file pone.0298949.s002.pdf]

Human Participants Research Checklist

Complete the following if your study involved human participants or human participants' data. These questions should be addressed for prospective and retrospective studies.

1. Did you obtain ethics approval for this study?
   - If yes, please upload (file type "Other") the original approval document you received from your ethics committee. <u>If the original document is in another language, please also provide an English translation.</u>

     ___ Uploaded ___

   - If you did not obtain ethical approval, please explain why this was not required below.

**NA**

2. If you prospectively recruited human participants for the study – for example, you conducted a clinical trial, distributed questionnaires, or obtained tissues, data or samples for the purposes of this study, please report in the Methods:
   i. the day, month and year of the **start and end** of the recruitment period for this study.
      i. Start Date: 09/24/2021
      ii. End Date: 02/07/2022
   ii. whether participants provided informed consent, and if so, what type was obtained (for instance, written or verbal, and if verbal, how it was documented and witnessed). If your study included minors, state whether you obtained consent from parents or guardians. If the need for consent was waived by the ethics committee, please include this information.
      i. Participants provided written consent during the enrollment process. Minors were not included in our study.

   ___ Completed ___

3. If you are reporting a retrospective study of medical records or archived samples, please report in the Methods section:
   i. the day, month and year when the data were accessed for research purposes
   ii. whether authors had access to information that could identify individual participants during or after data collection

   N/A

ORIGINAL IRB

November 13, 2019

JESSICA DONCKELS
DEPARTMENT OF PSYCHOLOGICAL SCIENCE

RE: UCI IRB HS# 2019-5153 *Holistic Stress Reduction through Multi-Modal Personal Chronicles*

The above-referenced human-subjects research project has been approved by the University of California, Irvine Institutional Review Board (UCI IRB). This approval is limited to the activities described in the approved Protocol Narrative, and extends to the performance of these activities at each respective site identified in the Application for IRB Review. In accordance with this approval, the specific conditions for the conduct of this research are listed below, and informed consent from subjects must be obtained unless otherwise indicated below. Additional conditions for the general conduct of human-subjects research are detailed on the attached sheet.

NOTE: Approval by the Institutional Review Board does not, in and of itself, constitute approval for the implementation of this research. Other institutional clearances and approvals may be required (e.g., EH&S, Radiation Safety, School Dean, other institutional IRBs). Research undertaken in conjunction with outside entities, such as drug or device companies, are typically contractual in nature and require an agreement between the University and the entity. Such agreements must be executed by an institutional official in Sponsored Projects, a division in the UCI Office of Research. The University is not obligated to legally defend or indemnify an employee who individually enters into these agreements and investigators are personally liable for contracts they sign. **Accordingly, the project should not begin until all required approvals have been obtained.**

Questions concerning the approval of this research project may be directed to the Office of Research, 141 Innovation Drive, Suite 250, Irvine, CA 92697-7600; 949-824-6068, 949-824-2125, or 949-824-0665 (biomedical committee) or 949-824-6662 (social-behavioral committee).

Expedited Review: Category 4 & 7

Susan Turner, Ph.D.,
Chair, Institutional Review Board

Approval Issued: 11/8/2019
Expiration Date: 11/7/2020
UCI (FWA) 00004071, Approved: January 31, 2003

IRB Determinations as Conditions of Approval:

Informed Consent Determinations:

1. Signed Informed Consent Required

APPROVAL CONDITIONS FOR ALL UCI HUMAN RESEARCH PROTOCOLS

POST-APPROVAL INVESTIGATOR RESPONSIBILITIES (PAIR):
In accordance with Federal regulations and HRP policies, there are Investigator responsibilities during the conduct, as well as after completion, of your research. Use the PAIR Worksheet to ensure adherence with your post-approval regulatory responsibilities.

UCI RESEARCH POLICIES:
All individuals engaged in human-subjects research are responsible for compliance with all applicable UCI Research Policies. The Lead Researcher (and Faculty Sponsor, if applicable) of the study is ultimately responsible for assuring all study team members adhere to applicable policies for the conduct of human-subjects research.

LEAD RESEARCHER (LR) RECORDKEEPING RESPONSIBILITIES:
LRs are responsible for the retention of protocol–related records. The following web pages should be reviewed for more information about the LR's recordkeeping responsibilities for the preparation and maintenance of research files: Lead Researcher Recordkeeping Responsibilities and Preparation and Maintenance of a Research Audit File.

APPROVED VERSIONS OF CONSENT DOCUMENTS, INCLUDING STUDY INFORMATION SHEETS:
Unless a waiver of informed consent is granted by the IRB, the consent documents (consent form; study information sheet) with the UCI IRB approval stamp must be used for consenting all human subjects enrolled in this study. Only the current approved version of the consent documents may be used to consent subjects. **Approved consent documents are not to be used beyond the expiration date provided on the IRB approval letter.** Current consent documents are available on the IRB Document Depot.

PROTOCOL EXPIRATION:
The UCI IRB approval letter references the protocol expiration date under the IRB Chair's signature authorization. A courtesy email will be sent approximately 60 to 90 days prior to expiration reminding the Lead Researcher to apply for continuing review**. It is the LR's responsibility to apply for continuing review to ensure continuing approval throughout the conduct of the study.** Lapses in approval must be avoided to protect the safety and welfare of enrolled subjects.

MODIFICATIONS & AMENDMENTS:
Per federal regulations, once a human research study has received IRB approval, any subsequent changes to the study must be reviewed and approved by the IRB prior to implementation *except when necessary to avoid an immediate, apparent hazard to a* subject. **Accordingly, no changes are permissible (*unless to avoid an immediate, apparent hazard to a subject*) to the approved protocol or the approved, stamped consent form without the prior review and approval of the UCI IRB**. All changes (e.g., a change in procedure, number of subjects, personnel, study locations, new recruitment materials, study instruments, etc.) must be prospectively reviewed and approved by the IRB before they are implemented.

CHANGES IN FINANCIAL INTEREST:
Any changes in the financial relationship between the study sponsor and any of the investigators on the study and/or any new potential conflicts of interest must be reported immediately to the UCI Conflict of Interest Oversight Committee (COIOC). If these changes affect the conduct of the study or result in a change in the text of the currently-approved informed consent document, these changes must also be reported to the UCI IRB via a modification request.

GRANT CONGRUENCE REVIEWS:
If this human subject research is funded or supported by a Federal Agency, it is the LR's responsibility to submit modifications, as necessary, to assure that the IRB protocol continues to be identical in principle and congruent with the scope of work outlined in the proposal application.

UNANTICIPATED PROBLEMS REPORTING:
In accordance with Federal regulations and HRP policies, only internal (where UCI serves as the IRB of record), Unanticipated Problems must be reported to the UCI IRB. Unanticipated Problems should also be reported to the UCI IRB when UCI is relying on an external IRB, and the incident occurred at UCI or the incident occurred at an offsite location on a study conducted by a UCI LR. Unanticipated Problems must be submitted to the IRB via the Unanticipated Problems (UP) Report within 5 business

days upon the LR’s knowledge of the event. For additional information visit the updated HPR webpage on Unanticipated Problems.

POSTING OF THE INFORMED CONSENT DOCUMENT:
Clinical trials initially approved by the IRB on or after January 21, 2019, must post one (1) IRB-approved clinical trial consent form at a publicly available federal website. The consent form must be posted after recruitment closes, and no later than 60 days after the last study visit. For additional guidance, refer to the OHRP FAQs on Informed Consent.

CLOSING REPORT:
A closing report should be filed with the UCI IRB when the research concludes. Visit the HRP webpage Closing a Protocol for complete details.

RENEWAL IRB

October 28, 2021

JESSICA DONCKELS
DEPARTMENT OF PSYCHOLOGICAL SCIENCE

RE: UCI IRB # 20195153 *Holistic Stress Reduction through Multi-Modal Personal Chronicles*

The above-referenced human-subjects research project has been approved by the University of California, Irvine Institutional Review Board (UCI IRB). In addition, an amendment has been approved. Specific changes approved by the IRB are noted below.

This approval is limited to the activities described in the approved protocol and extends to the performance of these activities at each respective site identified. In accordance with this approval, the specific conditions for the conduct of this research are listed below, and informed consent from subjects must be obtained unless otherwise indicated below. Additional conditions for the general conduct of human-subjects research are detailed on the attached sheet. If the approved amendment(s) includes changes to the informed consent document, the approved stamped consent form is enclosed. Please discontinue use of any previous versions of the informed consent document and use only the most updated version for enrollment of all new subjects.

NOTE: Approval by the Institutional Review Board does not, in and of itself, constitute approval for the implementation of this research. Other institutional clearances and approvals may be required. Research undertaken in conjunction with outside entities, such as drug or device companies, are typically contractual in nature and require an agreement between the University and the entity. Such agreements must be executed by an institutional official in Sponsored Projects, a division in the UCI Office of Research. The University is not obligated to legally defend or indemnify an employee who individually enters into these agreements and investigators are personally liable for contracts they sign. **Accordingly, the project should not begin until all required approvals have been obtained.**

Below is a summary of the approved changes requested:

Change in Research Procedure(s) (e.g., add MRI, or questionnaire, remove blood draw):
Participants will download an additional mobile app on to their phone called AWARE. This will be part of the suite of devices used that make-up PERSONICLE. In addition to the Personicle smartphone application, sensory data will also be collected through AWARE. AWARE is a framework dedicated to instrument, infer, log and share mobile context information by sensor instrumentation, for application developers, researchers and smartphone users. AWARE captures hardware-, software-, and human-based data from smartphones. For this study, we use the AWARE application and install it on participants' mobile devices to collect related context information to improve our accuracy to predict and intervene stress episodes. All identifiable or sensitive contents will not be collected, such as phone call contents, message contents, specific app usage, keyboard logs, phone numbers, names, passwords, etc. Below are the list of data collected through AWARE. Majority of these overlap with the PERSONICLE app, and are noted on the Protocol Narrative. ● Keyboard activity: speed of typing, frequency of using uppercase characters/numbers. Only the masked version of participants' text will be collected to our servers, the content of typed texts will not be collected/stored. The masking procedure entails replacing all lowercase characters with only one character and all uppercase characters with another character and all numbers with one number. So the collected text will consist of only 4/5 characters, allowing no information to be extracted from them. Keyboard activity is pertinent to study aims, as the frequency and pattern of keyboard usage can be a rich source of information about a person's communication behaviors and their level of connectivity with others. ● Social interactions via phone microphone: specifically, the number of people speaking detected by the phone microphone. No audio content or sensitive information will be collected or processed. This

sensor uses information processed by Android/IOS operating systems that could be obtained by any other application installed on a participant's phone and will only use the phone API (Application Programming Interface), which reports the number of people speaking in the environment. The current study is interested in these data because the number of people speaking in the participant's environment could provide insight into loneliness by tracking how many people participants are physically engaging with. ● Phone call information: time, duration, and type (received/sent/rejected) of phone calls. No content or audio from these phone calls will be collected or stored. Phone call information can not only indicate how often a person is in touch with others over the phone but how often they are reaching out, being reached out to, and/or isolating oneself. ● Notifications: Time, type, and source (i.e., application) of the notifications appearing on the participants phone. No notification content will be collected. The frequency and types of notifications received by a person could be an indicator of what types of apps they are interacting with most frequently. The reason for this additional mobile app and data collection is to strengthen the ability of PERSONICLE to take into consideration context in understanding college student mental health and behaviors. In collecting this additional data, we can better develop personalized models of mental health and intervention.

Change in Consent:
We are clarifying the language on the informed consent form. Participants will only be administered a mid-point assessment ONCE, after the 4-week monitoring period. We have edited the consent form language to accurately reflect this assessment schedule (specific wording changes are provided below for reference as well as updated on the protocol narrative). "After week 8, you will complete a similar battery of questionnaires as done at the pre-assessment (Baseline). At the end of the study (after the 12-week period), you will visit the lab to return the Personicle devices and complete similar questionnaires from the pre-assessment along with an exit interview regarding your experiences and attitudes with the devices."

Other Change(s):
We want to include 3 additional emotion-related measures to our study. This type of change does not qualify as adding a procedure and will allow the study to collect more dimensional, in-depth data regarding participants' emotional state and traits. Specifically, these will be added to the baseline assessment, the midpoint assessment, and the exit assessment. We make this edit inp the protocol narrative in section 6B (measures). First, we want to include the Implicit Beliefs about Emotions (De Castella et al., 2013) which is a measure about people's malleability beliefs about the malleability of emotions, or whether they are able to change how they feel. Secondly, we want to include a measure of emotional clarity (i.e., how much an individual knows what they are feeling) and attention to emotions (i.e., how much they notice their feelings). We are using a revised scale that combines items from the Trait-Meta Mood Scale (Salovey et al., 1995) and Toronto Alexithymia Scale (Bagby et al., 1994). The modified scale was created by Palmieri and colleagues (2009) who did a factor analysis to determine which items in both scales best reflected emotional clarity and attention to emotions. In summary, we hope to include assessments about people's beliefs about how much they can change their emotions, their emotional clarity, and attention to emotions. Our study involves participants completing longitudinal assessments of their positive and negative emotions throughout the day over the course of their participation in the study. While we are interested in how their daily emotions, physiological assessments, and behaviors may help us understand college student mental health, we are interested in traits that may relate to individual differences in the trajectory of their emotions or experiences. For example, a person's mind-set or health behaviors may be an important predictor in their emotions or activity. Emotion-traits may also be a predictor of mental health, future emotion/emotion in daily life, and behaviors. Thus, including assessments about how people believe or are aware of their emotions are important to consider.
Removed the following Research Personnel as study team has confirmed RPs are being kept track of on the Study Team Tracking Log: Asal Yunusova and Summer Millwood.

AMENDMENT NOTES: The IRB may not have approved all changes proposed in the amendment application. Review the above summary of approved changes and any revised documents provided with this letter. If a requested change does not appear in the summary or in the revised documents, the IRB did not approve that change. Please consult with Human Research Protections (HRP) Staff for further information. Unless

emergent and / or necessary to protect human subjects, changes to approved protocols may not be made without prior approval by the IRB.

Questions concerning the approval of this research project may be directed to the Office of Research, 160 Aldrich Hall, Irvine, CA 92697-7600; 949-824-6068, 949-824-2125, or 949-824-0665 (biomedical committee) or 949-824-6662 (social-behavioral committee).

Expedited Review: Categories 4, 7: October 28, 2021

Susan Turner, Ph.D.,
Vice-Chair, Institutional Review Board

Approval Issued: 10/28/2021
Expiration Date: 10/27/2024
UCI (FWA) 00004071, Approved: January 31, 2003

Important Reminder: **UCI is in Research Phase 4 as of June 22, 2021. UCI's research activities will increase over time in parallel with the stages in California's Pandemic Roadmap and other public health and higher education guidance. Refer to the Office of Research webpage on Research Continuity for more details.**

IRB Determinations as Conditions of Approval:
Study Status:
1. Three-Year Extended IRB Approval Granted[1]

Informed Consent Determinations:
2. Signed Informed Consent Required

[1] *Pre-2018 Common Rule Only: Research posing no more than minimal risk to human subjects (Expedited review) and is not subject to federal oversight (e.g. federally-supported) qualifies for Extended IRB Approval. If during the extended approval period the study becomes ineligible for Extended IRB Approval immediately contact the HRP staff for instructions on how to reset to a one-year (no more than 365 days) approval cycle.*

APPROVAL CONDITIONS FOR ALL UCI HUMAN RESEARCH PROTOCOLS

POST-APPROVAL INVESTIGATOR RESPONSIBILITIES (PAIR):
In accordance with Federal regulations and HRP policies, there are Investigator responsibilities during the conduct, as well as after completion, of your research. Use the PAIR Worksheet to ensure adherence with your post-approval regulatory responsibilities.

UCI RESEARCH POLICIES:
All individuals engaged in human-subjects research are responsible for compliance with all applicable UCI Research Policies. The Lead Researcher (and Faculty Sponsor, if applicable) of the study is ultimately responsible for assuring all study team members adhere to applicable policies for the conduct of human-subjects research.

LEAD RESEARCHER (LR) RECORDKEEPING RESPONSIBILITIES:
LRs are responsible for the retention of protocol–related records. The following web pages should be reviewed for more information about the LR's recordkeeping responsibilities for the preparation and maintenance of research files: Lead Researcher Recordkeeping Responsibilities and Preparation and Maintenance of a Research Audit File.

APPROVED VERSIONS OF CONSENT DOCUMENTS, INCLUDING STUDY INFORMATION SHEETS:
Unless a waiver of informed consent is granted by the IRB, the consent documents (consent form; study information sheet) with the UCI IRB approval stamp must be used for consenting all human subjects enrolled in this study. Only the current approved version of the consent documents may be used to consent subjects. **Approved consent documents are not to be used beyond the expiration date provided on the IRB approval letter.** IRB approved materials can be found in KR Protocols in the attachments section.

PROTOCOL EXPIRATION:
The UCI IRB approval letter references the protocol expiration date under the IRB Chair's signature authorization. A courtesy email will be sent prior to expiration reminding the Lead Researcher to apply for renewal**. It is the LR's responsibility to apply for renewal to ensure continuous approval throughout the conduct of the study.** Lapses in approval must be avoided to protect the safety and welfare of enrolled subjects.

AMENDMENTS:
The UCI HRP does not require the submission of minor changes to exempt research. For those studies in which a lead researcher (and faculty sponsor (FS), if applicable) has submitted to and received UCI IRB confirmation of exemption, minor changes may be made without notifying the UCI IRB. For more information about this including what constitutes a minor change versus a change that must be prospectively submitted for review and approval by the UCI IRB via a formal amendment, visit Protocol Amendment.

CHANGES IN FINANCIAL INTEREST:
Any changes in the financial relationship between the study sponsor and any of the investigators on the study and/or any new potential conflicts of interest must be reported immediately to the UCI Conflict of Interest Oversight Committee (COIOC). If these changes affect the conduct of the study or result in a change in the text of the approved informed consent document, these changes must also be reported to the UCI IRB via an amendment.

GRANT CONGRUENCE REVIEWS:
If this human subject research is funded or supported by a Federal Agency, it is the LR's responsibility to submit amendments, as necessary, to assure that the IRB protocol continues to be identical in principle and congruent with the scope of work outlined in the proposal application.

REPORTING A PROBLEM:

In accordance with Federal regulations and HRP policies, only internal (where UCI serves as the IRB of record), Unanticipated Problems must be reported to the UCI IRB. Unanticipated Problems should also be reported to the UCI IRB when UCI is relying on an external IRB, and the incident occurred at UCI or the incident occurred at an offsite location on a study conducted by a UCI LR. Unanticipated Problems must be submitted to the IRB within 5 business days upon the LR's knowledge of the event. For additional information visit the updated HPR webpage on Reporting a Problem.

POSTING OF THE INFORMED CONSENT DOCUMENT:

Clinical trials initially approved by the IRB on or after January 21, 2019, must post one (1) IRB-approved clinical trial consent form at a publicly available federal website. The consent form must be posted after recruitment closes, and no later than 60 days after the last study visit. For additional guidance, refer to the OHRP FAQs on Informed Consent.

CLOSING REPORT:

A closing report should be filed with the UCI IRB when the research concludes. Visit the HRP webpage Closing a Protocol for complete details.
