# Supplementary material for: Objective monitoring of loneliness levels using smart devices: A multi-device approach for mental health applications
Source: PLoS One. 2024 Jun 20;19(6):e0298949. doi: 10.1371/journal.pone.0298949 (PMC11189241; doi:10.1371/journal.pone.0298949)
Supplement: Attachment — Submitted filename: PLOS Human Participants Research Checklist.pdf [file pone.0298949.s003.pdf]

Human Participants Research Checklist

Complete the following if your study involved human participants or human participants' data. These questions should be addressed for prospective and retrospective studies.

1. Did you obtain ethics approval for this study?

   - If yes, please upload (file type "Other") the original approval document you received from your ethics committee. <u>If the original document is in another language, please also provide an English translation.</u>

     ___ Uploaded ___

   - If you did not obtain ethical approval, please explain why this was not required below.

NA

2. If you prospectively recruited human participants for the study – for example, you conducted a clinical trial, distributed questionnaires, or obtained tissues, data or samples for the purposes of this study, please report in the Methods:

   i. the day, month and year of the **start and end** of the recruitment period for this study.

      i. Start Date: 09/24/2021

      ii. End Date: 02/07/2022

   ii. whether participants provided informed consent, and if so, what type was obtained (for instance, written or verbal, and if verbal, how it was documented and witnessed). If your study included minors, state whether you obtained consent from parents or guardians. If the need for consent was waived by the ethics committee, please include this information.

      i. Participants provided written consent during the enrollment process. Minors were not included in our study.

   ___ Completed ___

3. If you are reporting a retrospective study of medical records or archived samples, please report in the Methods section:

   i. the day, month and year when the data were accessed for research purposes

   ii. whether authors had access to information that could identify individual participants during or after data collection

   N/A

ORIGINAL IRB

November 13, 2019

JESSICA DONCKELS
DEPARTMENT OF PSYCHOLOGICAL SCIENCE

RE: UCI IRB HS# 2019-5153 *Holistic Stress Reduction through Multi-Modal Personal Chronicles*

The above-referenced human-subjects research project has been approved by the University of California, Irvine Institutional Review Board (UCI IRB). This approval is limited to the activities described in the approved Protocol Narrative, and extends to the performance of these activities at each respective site identified in the Application for IRB Review. In accordance with this approval, the specific conditions for the conduct of this research are listed below, and informed consent from subjects must be obtained unless otherwise indicated below. Additional conditions for the general conduct of human-subjects research are detailed on the attached sheet.

NOTE: Approval by the Institutional Review Board does not, in and of itself, constitute approval for the implementation of this research. Other institutional clearances and approvals may be required (e.g., EH&S, Radiation Safety, School Dean, other institutional IRBs). Research undertaken in conjunction with outside entities, such as drug or device companies, are typically contractual in nature and require an agreement between the University and the entity. Such agreements must be executed by an institutional official in Sponsored Projects, a division in the UCI Office of Research. The University is not obligated to legally defend or indemnify an employee who individually enters into these agreements and investigators are personally liable for contracts they sign. **Accordingly, the project should not begin until all required approvals have been obtained.**

Questions concerning the approval of this research project may be directed to the Office of Research, 141 Innovation Drive, Suite 250, Irvine, CA 92697-7600; 949-824-6068, 949-824-2125, or 949-824-0665 (biomedical committee) or 949-824-6662 (social-behavioral committee).

Expedited Review: Category 4 & 7

Susan Turner, Ph.D.,
Chair, Institutional Review Board

Approval Issued: 11/8/2019
Expiration Date: 11/7/2020
UCI (FWA) 00004071, Approved: January 31, 2003

IRB Determinations as Conditions of Approval:

Informed Consent Determinations:
1. Signed Informed Consent Required

APPROVAL CONDITIONS FOR ALL UCI HUMAN RESEARCH PROTOCOLS

POST-APPROVAL INVESTIGATOR RESPONSIBILITIES (PAIR):
In accordance with Federal regulations and HRP policies, there are Investigator responsibilities during the conduct, as well as after completion, of your research. Use the PAIR Worksheet to ensure adherence with your post-approval regulatory responsibilities.

UCI RESEARCH POLICIES:
All individuals engaged in human-subjects research are responsible for compliance with all applicable UCI Research Policies. The Lead Researcher (and Faculty Sponsor, if applicable) of the study is ultimately responsible for assuring all study team members adhere to applicable policies for the conduct of human-subjects research.

LEAD RESEARCHER (LR) RECORDKEEPING RESPONSIBILITIES:
LRs are responsible for the retention of protocol–related records. The following web pages should be reviewed for more information about the LR's recordkeeping responsibilities for the preparation and maintenance of research files: Lead Researcher Recordkeeping Responsibilities and Preparation and Maintenance of a Research Audit File.

APPROVED VERSIONS OF CONSENT DOCUMENTS, INCLUDING STUDY INFORMATION SHEETS:
Unless a waiver of informed consent is granted by the IRB, the consent documents (consent form; study information sheet) with the UCI IRB approval stamp must be used for consenting all human subjects enrolled in this study. Only the current approved version of the consent documents may be used to consent subjects. **Approved consent documents are not to be used beyond the expiration date provided on the IRB approval letter.** Current consent documents are available on the IRB Document Depot.

PROTOCOL EXPIRATION:
The UCI IRB approval letter references the protocol expiration date under the IRB Chair's signature authorization. A courtesy email will be sent approximately 60 to 90 days prior to expiration reminding the Lead Researcher to apply for continuing review**. It is the LR's responsibility to apply for continuing review to ensure continuing approval throughout the conduct of the study.** Lapses in approval must be avoided to protect the safety and welfare of enrolled subjects.

MODIFICATIONS & AMENDMENTS:
Per federal regulations, once a human research study has received IRB approval, any subsequent changes to the study must be reviewed and approved by the IRB prior to implementation *except when necessary to avoid an immediate, apparent hazard to a* subject. **Accordingly, no changes are permissible (*unless to avoid an immediate, apparent hazard to a subject*) to the approved protocol or the approved, stamped consent form without the prior review and approval of the UCI IRB**. All changes (e.g., a change in procedure, number of subjects, personnel, study locations, new recruitment materials, study instruments, etc.) must be prospectively reviewed and approved by the IRB before they are implemented.

CHANGES IN FINANCIAL INTEREST:
Any changes in the financial relationship between the study sponsor and any of the investigators on the study and/or any new potential conflicts of interest must be reported immediately to the UCI Conflict of Interest Oversight Committee (COIOC). If these changes affect the conduct of the study or result in a change in the text of the currently-approved informed consent document, these changes must also be reported to the UCI IRB via a modification request.

GRANT CONGRUENCE REVIEWS:
If this human subject research is funded or supported by a Federal Agency, it is the LR's responsibility to submit modifications, as necessary, to assure that the IRB protocol continues to be identical in principle and congruent with the scope of work outlined in the proposal application.

UNANTICIPATED PROBLEMS REPORTING:
In accordance with Federal regulations and HRP policies, only internal (where UCI serves as the IRB of record), Unanticipated Problems must be reported to the UCI IRB. Unanticipated Problems should also be reported to the UCI IRB when UCI is relying on an external IRB, and the incident occurred at UCI or the incident occurred at an offsite location on a study conducted by a UCI LR. Unanticipated Problems must be submitted to the IRB via the Unanticipated Problems (UP) Report within 5 business

days upon the LR's knowledge of the event. For additional information visit the updated HPR webpage on Unanticipated Problems.

POSTING OF THE INFORMED CONSENT DOCUMENT:

Clinical trials initially approved by the IRB on or after January 21, 2019, must post one (1) IRB-approved clinical trial consent form at a publicly available federal website. The consent form must be posted after recruitment closes, and no later than 60 days after the last study visit. For additional guidance, refer to the OHRP FAQs on Informed Consent.

CLOSING REPORT:

A closing report should be filed with the UCI IRB when the research concludes. Visit the HRP webpage Closing a Protocol for complete details.

RENEWAL IRB

UCI University of California, Irvine

October 10, 2020

JESSICA DONCKELS
DEPARTMENT OF PSYCHOLOGICAL SCIENCE

RE: UCI IRB HS# 2019-5153 *Holistic Stress Reduction through Multi-Modal Personal Chronicles*

The above-referenced human-subjects research project has been approved by the University of California, Irvine Institutional Review Board (UCI IRB). This approval is limited to the activities described in the approved Protocol Narrative, and extends to the performance of these activities at each respective site identified in the Application for IRB Review. In accordance with this approval, the specific conditions for the conduct of this research are listed below, and informed consent from subjects must be obtained unless otherwise indicated below. Additional conditions for the general conduct of human-subjects research are detailed on the attached sheet.

NOTE: Approval by the Institutional Review Board does not, in and of itself, constitute approval for the implementation of this research. Other institutional clearances and approvals may be required (e.g., EH&S, Radiation Safety, School Dean, other institutional IRBs). Research undertaken in conjunction with outside entities, such as drug or device companies, are typically contractual in nature and require an agreement between the University and the entity. Such agreements must be executed by an institutional official in Sponsored Projects, a division in the UCI Office of Research. The University is not obligated to legally defend or indemnify an employee who individually enters into these agreements and investigators are personally liable for contracts they sign. **Accordingly, the project should not begin until all required approvals have been obtained.**

Questions concerning the approval of this research project may be directed to the Office of Research, 141 Innovation Drive, Suite 250, Irvine, CA 92697-7600; 949-824-6068, 949-824-2125, or 949-824-0665 (biomedical committee) or 949-824-6662 (social-behavioral committee).

Expedited Review: Categoryies 4,7

Elizabeth Cauffman, Ph.D.,
Chair, Institutional Review Board

Approval Issued: 10/29/2020
Expiration Date: 11/1/2021
UCI (FWA) 00004071, Approved: January 31, 2003

Important Reminder: UCI is in Research Phase 2 as of June 8, 2020. UCI's research activities will increase over time in parallel with the stages in California's Pandemic Roadmap and other public health and higher education guidance. Refer to the Office of Research webpage on Research Continuity for more details.

IRB Determinations as Conditions of Approval:

Informed Consent Determinations:

1. Signed Informed Consent Required

APPROVAL CONDITIONS FOR ALL UCI HUMAN RESEARCH PROTOCOLS

POST-APPROVAL INVESTIGATOR RESPONSIBILITIES (PAIR):
In accordance with Federal regulations and HRP policies, there are Investigator responsibilities during the conduct, as well as after completion, of your research. Use the PAIR Worksheet to ensure adherence with your post-approval regulatory responsibilities.

UCI RESEARCH POLICIES:
All individuals engaged in human-subjects research are responsible for compliance with all applicable UCI Research Policies. The Lead Researcher (and Faculty Sponsor, if applicable) of the study is ultimately responsible for assuring all study team members adhere to applicable policies for the conduct of human-subjects research.

LEAD RESEARCHER (LR) RECORDKEEPING RESPONSIBILITIES:
LRs are responsible for the retention of protocol–related records. The following web pages should be reviewed for more information about the LR's recordkeeping responsibilities for the preparation and maintenance of research files: Lead Researcher Recordkeeping Responsibilities and Preparation and Maintenance of a Research Audit File.

APPROVED VERSIONS OF CONSENT DOCUMENTS, INCLUDING STUDY INFORMATION SHEETS:
Unless a waiver of informed consent is granted by the IRB, the consent documents (consent form; study information sheet) with the UCI IRB approval stamp must be used for consenting all human subjects enrolled in this study. Only the current approved version of the consent documents may be used to consent subjects. **Approved consent documents are not to be used beyond the expiration date provided on the IRB approval letter.** Current consent documents are available on the IRB Document Depot.

PROTOCOL EXPIRATION:
The UCI IRB approval letter references the protocol expiration date under the IRB Chair's signature authorization. A courtesy email will be sent approximately 60 to 90 days prior to expiration reminding the Lead Researcher to apply for continuing review**. It is the LR's responsibility to apply for continuing review to ensure continuing approval throughout the conduct of the study.** Lapses in approval must be avoided to protect the safety and welfare of enrolled subjects.

MODIFICATIONS & AMENDMENTS:
Per federal regulations, once a human research study has received IRB approval, any subsequent changes to the study must be reviewed and approved by the IRB prior to implementation *except when necessary to avoid an immediate, apparent hazard to a* subject. **Accordingly, no changes are permissible (*unless to avoid an immediate, apparent hazard to a subject*) to the approved protocol or the approved, stamped consent form without the prior review and approval of the UCI IRB**. All changes (e.g., a change in procedure, number of subjects, personnel, study locations, new recruitment materials, study instruments, etc.) must be prospectively reviewed and approved by the IRB before they are implemented.

CHANGES IN FINANCIAL INTEREST:
Any changes in the financial relationship between the study sponsor and any of the investigators on the study and/or any new potential conflicts of interest must be reported immediately to the UCI Conflict of Interest Oversight Committee (COIOC). If these changes affect the conduct of the study or result in a change in the text of the currently-approved informed consent document, these changes must also be reported to the UCI IRB via a modification request.

GRANT CONGRUENCE REVIEWS:
If this human subject research is funded or supported by a Federal Agency, it is the LR's responsibility to submit modifications, as necessary, to assure that the IRB protocol continues to be identical in principle and congruent with the scope of work outlined in the proposal application.

UNANTICIPATED PROBLEMS REPORTING:
In accordance with Federal regulations and HRP policies, only internal (where UCI serves as the IRB of record), Unanticipated Problems must be reported to the UCI IRB. Unanticipated Problems should also be reported to the UCI IRB when UCI is relying on an external IRB, and the incident occurred at UCI or the incident occurred at an offsite location on a study conducted by a UCI LR. Unanticipated Problems must be submitted to the IRB via the Unanticipated Problems (UP) Report within 5 business

days upon the LR's knowledge of the event. For additional information visit the updated HPR webpage on Unanticipated Problems.

POSTING OF THE INFORMED CONSENT DOCUMENT:

Clinical trials initially approved by the IRB on or after January 21, 2019, must post one (1) IRB-approved clinical trial consent form at a publicly available federal website. The consent form must be posted after recruitment closes, and no later than 60 days after the last study visit. For additional guidance, refer to the OHRP FAQs on Informed Consent.

CLOSING REPORT:

A closing report should be filed with the UCI IRB when the research concludes. Visit the HRP webpage Closing a Protocol for complete details.
